# Isolation and biocontrol potential of *Bacillus amyloliquefaciens* against asparagus stem blight

**DOI:** 10.3389/fmicb.2026.1872041

**Published:** 2026-07-09

**Authors:** Ke-Li Jia, Ping-Fa Li, Rui-Dong Wen, Wu-Jun Gao, Shu-Fen Li

**Affiliations:** 1 School of Medical Laboratory, North Henan Medical University, Xinxiang, Henan, China; 2 College of Life Sciences, Henan Normal University, Xinxiang, China

**Keywords:** *Bacillus amyloliquefaciens*, biological control, garden asparagus, *Phomopsis asparagi*, stem blight

## Abstract

Garden asparagus (*Asparagus officinalis* L.) is an economically important vegetable crop widely cultivated for its nutritional value and health-promoting properties. Asparagus stem blight, caused by *Phomopsis asparagi* (Sacc.) Bubák, is one of the most destructive diseases affecting asparagus production worldwide, leading to significant yield losses. In this study, a pathogenic strain of *P. asparagi* was isolated and characterized, serving as the target for screening antagonistic microorganisms from the rhizosphere soil of healthy asparagus plants. Among 79 isolates, one strain, identified as *Bacillus amyloliquefaciens* HN2-9 based on morphological, physiological, and molecular analyses, exhibited the strongest inhibitory activity against the pathogen. HN2-9 caused hyphal deformation, fragmentation, and lysis of *P. asparagi*. Optimal growth and fermentation conditions were established, achieving a maximum hyphal inhibition rate of 92.1% and spore germination inhibition of 54.8%. In greenhouse assays, HN2-9 significantly reduced disease severity on asparagus stems, with a disease index decrease of 72.4% at OD₆₀₀ = 2, without affecting seed germination or plant growth. These results demonstrate that *B. amyloliquefaciens* HN2-9 is an effective biocontrol agent against asparagus stem blight and provide a foundation for its development as a sustainable, environmentally friendly disease management strategy.

## Introduction

Garden asparagus (*Asparagus officinalis* L.) is a perennial vegetable crop of the family Asparagaceae, valued for its high nutritional content, health-promoting compounds, and desirable sensory qualities, making it an economically important crop worldwide (Yang et al., 2020). However, asparagus production is severely constrained by fungal diseases, particularly stem blight, commonly referred to as the “cancer” of asparagus, which has a severe impact on both yield and quality (Yang et al., 2016). This disease, caused by *Phomopsis asparagi* (Sacc.) Bubák, is widely distributed across major asparagus-producing regions, including Asia, North America, Africa, Europe, and southern Australia (Elena, 2006; Iwato et al., 2014; McKirdy et al., 2002; Thao and Dung, 2019; Zaw et al., 2017), with especially severe epidemics occurring in Asian countries (Zhang and Araki, 2017).

The pathogen *P. asparagi* is a hemibiotrophic filamentous fungus belonging to the family Sphaeropsidaceae. Its asexual spores (conidia) are predominantly *α*-type, elongated and containing one to two oil droplets, whereas *β*-type and intermediate forms occur less frequently (Yang et al., 2016). In the field, the fungus overwinters in infected plant debris and soil, releasing conidia under humid conditions to infect emerging asparagus stems. Disease development is strongly influenced by environmental factors, including temperature, rainfall, soil moisture, and cultivation practices, with optimal infection occurring at 26 °C–28 °C and visible symptoms appearing within 5–7 days after infection. Typical symptoms begin as water-soaked lesions that expand into deep brown, spindle-shaped spots, ultimately leading to stem desiccation and plant death.

At the cellular level, *P. asparagi* initiates infection by rapid conidial germination on the stem surface, followed by host penetration via specialized infection structures and subsequent colonization of epidermal and cortical tissues. The pathogen exhibits both intercellular and intracellular hyphal growth, facilitating extensive tissue invasion and cellular damage. As infection progresses, hyphae penetrate deeper stem tissues, culminating in pycnidia formation and conidial release, thereby enabling secondary infection cycles. In regions with mild winters, such repeated infection cycles can occur annually, further complicating disease management (Sun et al., 2024).

Currently, asparagus stem blight control relies primarily on chemical fungicides, which, although effective, pose environmental risks, increase production costs, and may lead to the development of resistant pathogen strains (Shi et al., 2020). These limitations highlight the urgent need for sustainable and environmentally friendly disease management strategies. Biological control, using beneficial microorganisms to inhibit pathogen growth or induce plant resistance, offers a promising alternative to chemical fungicides. Rhizosphere soil-associated microorganisms, such as bacteria, fungi, actinomycetes, and yeasts, not only directly suppress plant pathogens through producing antibiotics, lytic enzymes, siderophores, and other secondary metabolites, but also indirectly enhance plant resistance through induced systemic resistance or other host defense pathways, making them sustainable components of integrated disease management strategies (Liu et al., 2025; Zehra et al., 2021).

Among biological control agents, bacteria are the most widely used due to their robust environmental tolerance and versatile mechanisms of pathogen suppression. Members of the genus *Bacillus* are particularly prominent as biocontrol agents because they can efficiently colonize plant roots, produce diverse bioactive compounds and enhance plant defense through direct inhibition and competition with pathogens (Etesami et al., 2023; Karačić et al., 2024). In practical applications, different species and strains of *Bacillus* spp. have been successfully applied to control diseases in various crops, such as rice, chili, apple, lettuce, cotton, and kiwifruit (Aziz et al., 2024; Bahadou et al., 2018; Petkova and Dimova, 2024; Rais et al., 2017; Sampathkumar et al., 2023; Wang et al., 2023). For example, endophytic *Bacillus* strains isolated from cotton roots strongly inhibited *Verticillium dahliae* hyphal growth, causing shrinkage and deformation, while also enhancing host defense gene expression and significantly reducing disease incidence (Nadeem et al., 2020).

Despite advances in understanding bacterial antagonists, research on biological control of asparagus stem blight caused by *P. asparagi* remains very limited, and only a small number of microbial agents have been evaluated for their effectiveness against this disease. Furthermore, little information is available regarding the optimization of culture conditions and practical application potential of antagonistic microorganisms targeting *P. asparagi*. The present study aims to isolate and identify a potent biocontrol bacterium from the rhizosphere of healthy asparagus plants, optimize its growth and fermentation conditions, and characterize its antagonistic activity against *P. asparagi*. A novel strain, *Bacillus amyloliquefaciens* HN2-9, was identified and comprehensively evaluated for its inhibitory effects on mycelial growth and spore germination of *P. asparagi*, as well as its disease suppression efficacy under greenhouse conditions. By integrating strain screening, fermentation optimization, and greenhouse validation, this study provides new insights into the biological control of asparagus stem blight and expands the repertoire of potential microbial resources for sustainable disease management in asparagus production.

## Materials and methods

### Pathogen isolation and identification

Infected asparagus stems showing typical stem blight symptoms were collected from experimental fields. Stem segments were surface-sterilized sequentially with 1% NaClO, 75% (v/v) ethanol, and sterile water, then placed on PSA solid medium and incubated at 25 °C. Emerging fungal colonies were transferred to fresh PSA plates for purification. Morphological characteristics were examined using light microscopy after spore germination. For detailed infection observation, asparagus stems were inoculated with a spore suspension (1 × 10^7^ spores/mL), maintained under controlled humidity and temperature, and sampled at 3–4 days post-inoculation. Samples were fixed, dehydrated, and prepared for scanning electron microscopy and confocal laser scanning microscopy using standard protocols, including tissue clearing and fluorescent staining with WGA-AF488 and PI. For molecular identification, fungal DNA was extracted using a commercial kit, the internal transcribed spacer (ITS) region was sequenced, and sequence was compared against the NCBI database. The ITS sequence has been deposited in the NCBI GenBank database under accession number PZ556901. To further assess the reliability of species identification, TEF1-α and CAL sequences retrieved from an unpublished genome assembly of isolate ZF-C were compared with reference sequences from other related species. Phylogenetic analysis was performed using IQtree (Minh et al., 2020) to determine the relationship of the isolated pathogen. Furthermore, to confirm the pathogenicity of the isolated fungus, fungal cultures grown on PSA plates were inoculated onto healthy asparagus stems. Symptomatic tissues were subsequently collected, and the pathogen was re-isolated for comparison with the original isolate.

### Isolation of rhizosphere-associated bacteria

Rhizosphere soil was collected from healthy asparagus plants in diseased fields in Huixian, Xinxiang, Henan Province, China. Soil samples were air-dried, ground, serially diluted, and spread onto LB agar plates, followed by incubation at 28 °C for 3–7 days. Distinct single colonies were selected and purified by repeated streaking.

### Screening of antagonistic bacteria

Antagonistic activity against the asparagus stem blight pathogen was evaluated using a dual-culture assay on PSA medium. Activated bacterial cultures were inoculated at four symmetrical positions around a central pathogen mycelial plug, with sterile water used as the control. Plates were incubated at 25 °C for 5–7 days, and inhibition rates were calculated based on mycelial growth reduction.

### Identification of strain HN2-9

The antagonistic effect of strain HN2-9 was further examined by light microscopy and scanning electron microscopy using hyphae collected from the inhibition zone. Colony morphology and Gram staining were used for preliminary characterization. Physiological and biochemical properties were assessed under different pH and NaCl conditions and using the API 50CH system. For molecular identification, genomic DNA was extracted and the 16S rDNA and gyrA genes were amplified and sequenced. Homologous sequences were aligned, trimmed, and phylogenetic trees were constructed using IQtree. The 16S rDNA and gyrA sequences have been deposited in the NCBI GenBank database under accession number PZ556894 and PZ569087.

### Antagonistic effects of strain HN2-9 on pathogen hyphae

A dual-culture assay was performed by placing a mycelial plug of the pathogen on one side of PSA plates and inoculating 1 μL of HN2-9 suspension on the opposite side, with sterile water as the control. Plates were incubated at 25 °C for 7 days. Hyphae from the inhibition zone were collected and observed under a light microscope, and ultrastructural changes were further examined using scanning electron microscopy following standard sample preparation procedures.

### Growth curve and growth condition optimization of strain HN2-9

The growth curve of strain HN2-9 was determined in LB and NA liquid media using a 96-well microplate. Each treatment well contained 198 μL of medium inoculated with 2 μL of activated bacterial culture. Control wells contained 198 μL of uninoculated medium and 2 μL sterile water. OD_600_ values were recorded every 2 h for 96 h using an automated growth curve analyzer.

To optimize growth conditions, single-factor experiments were conducted by varying incubation temperature (30 °C–40 °C), initial pH (5–9), inoculum size (1–11%, v/v, seed culture volume ratio), and culture volume (50–200 mL). Cultures were incubated at 200 r/min for 24 h, and bacterial growth was evaluated by measuring OD_600_.

### Preparation of fermentation filtrate and antifungal activity assay

Strain HN2-9 was inoculated at 1% (v/v) into 100 mL LB broth using a seed culture adjusted to OD_600_ = 2.0, corresponding to approximately 3.3 × 10^8^ CFU/mL as determined by plate counting. The culture was incubated at 30 °C with shaking at 200 r/min for 5 days. The fermentation broth was centrifuged at 12,000 r/min for 10 min, and the supernatant was filtered through a 0.22-μm membrane to obtain the fermentation filtrate.

Antifungal activity was evaluated using an amended medium assay by mixing the fermentation filtrate with molten PSA medium, with sterile water serving as the control. A mycelial plug of the pathogen was placed at the center of each plate and incubated at 25 °C. Inhibition rates were calculated based on mycelial growth reduction.

### Optimization of fermentation conditions of strain HN2-9

Single-factor experiments were conducted to optimize fermentation conditions, including inoculum size (1–13%, v/v), fermentation time (1–7 days), initial pH (5–9), incubation temperature (30 °C–40 °C), and culture volume (50–200 mL). Fermentation filtrates obtained under each condition were prepared and their antifungal activities were evaluated using the amended medium assay.

### Optimization of fermentation medium components

The effects of different fermentation medium components on antifungal activity were evaluated by substituting individual components of LB medium. Carbon sources, nitrogen sources, and inorganic salts were tested separately. Based on single-factor results, an orthogonal experimental design was applied to determine the optimal combination of key medium components.

### Effect of fermentation filtrate on spore germination

Equal volumes of pathogen spore suspension and fermentation filtrate were mixed and incubated in PSA liquid medium at 25 °C, with sterile water as the control. Spore germination was observed microscopically at multiple time points, and germination rates were calculated.

### Effects of HN2-9 treatments on asparagus seed germination

Asparagus seeds were surface-sterilized with 1% NaClO followed by 75% ethanol and rinsed with sterile water. Fifty uniform seeds per treatment were soaked for 5 h in HN2-9 fermentation broth, fermentation filtrate, 10^−1^ or 10^−2^ diluted filtrates, or sterile water as the control. Seeds were then placed in sterile Petri dishes under moist conditions, and germination rates were recorded after 5–10 days. Each treatment was performed with three replicates.

### Control effect of strain HN2-9 on asparagus stem blight

Based on the results of *in vitro* antifungal assays using cell-free culture filtrates, culture conditions were optimized, and bacterial suspensions used for subsequent plant assays were prepared under these optimized conditions to ensure maximal antagonistic potential. Activated cultures of strain HN2-9 were grown to OD_600_ values of 0.8, 1.6, and 2.0. The viable cell concentration corresponding to OD_600_ = 2.0 was determined by standard plate counting and was 3.3 × 10^8^ CFU/mL, which served as a reference for bacterial density under the applied culture conditions. The bacterial suspensions at different OD_600_ levels were used directly from culture without washing or resuspension. Healthy asparagus shoots with uniform growth were selected. Filter paper strips (1 × 1 cm) were inoculated with 0.5 mL of pathogen spore suspension (1 × 10^7^ spores/mL), together with 0.5 mL of HN2-9 culture at the corresponding OD_600_ level. Treatments with pathogen alone, HN2-9 alone, and uninoculated liquid medium (the optimized fermentation medium) were served as controls. The filter papers were attached to asparagus stems approximately 2 cm above the soil surface and secured with grafting film. Incubated plants were grown at 25 °C and 90% relative humidity for 3 days. After removal of the filter papers, plants were maintained at 25 °C and 60% relative humidity. Disease symptoms were evaluated at 7 days post-inoculation by measuring lesion length, and disease index and control efficacy were calculated. Each treatment included five shoots and three biological replicates.

### Statistical analysis

Experiments involving quantitative measurements were performed with at least three biological replicates. Data are presented as mean ± standard deviation (SD). Statistical significance was determined using one-way analysis of variance (ANOVA) followed by Tukey’s multiple comparison test, with *p* < 0.05 considered statistically significant.

## Results

### Isolation and identification of the causal agent of asparagus stem blight

A fungal pathogen was isolated from symptomatic asparagus stems (Supplementary Figure S1A). The isolate was subcultured and designated as ZF-C. During early growth, colonies exhibited dense white aerial hyphae (Supplementary Figure S1B), which gradually turned greenish-brown with pigment accumulation at later stages (Supplementary Figure S1C). Black, globose pycnidia were subsequently formed and ruptured to release conidial masses (Supplementary Figure S1D).

Microscopic observation revealed that most conidia are fusiform and contain two oil droplets, consistent with *α*-type conidia (Supplementary Figure S1E). Germination of conidia typically occurred from one or both ends or occasionally from the middle, followed by branched, dendritic hyphal growth (Supplementary Figure S1F). Scanning electron microscopy further showed that after germination on asparagus stems, hyphae adhered to the stem surface and spread along the intercellular spaces of epidermal tissues in a dendritic pattern (Supplementary Figure S1G). After penetration, hyphae extended progressively along the inner walls of host cells, forming multilayered invasive structures within the epidermal tissues (Supplementary Figure S1H).

For molecular identification, the ITS region of isolate ZF-C was amplified and sequenced, yielding a fragment of 548 bp. BLAST analysis against GenBank revealed high sequence similarity to *P. asparagi*. Phylogenetic analysis based on ITS sequences showed that isolate ZF-C clusters most closely with *P. asparagi* isolate L24 (MN089630.1), with a sequence identity of 99.82% (Supplementary Figure S1I). Furthermore, phylogenetic analyses based on the TEF-1α and CAL gene sequences yielded consistent results (Supplementary Figures S1J,S1K).

To confirm pathogenicity, healthy asparagus stems were inoculated with isolate ZF-C, which reproduced typical stem blight symptoms. The pathogen was subsequently re-isolated from symptomatic tissues, and the recovered isolate showed morphological characteristics consistent with the original isolate, thereby fulfilling Koch’s postulates.

Taken together, based on Koch’s postulates, colony morphology, microscopic characteristics, infection behavior on host tissues, and molecular phylogenetic analysis, the isolated pathogen was identified as *P. asparagi*, the causal agent of asparagus stem blight.

### Isolation, screening and identification of antagonistic bacteria against asparagus stem blight

**Figure 1:**
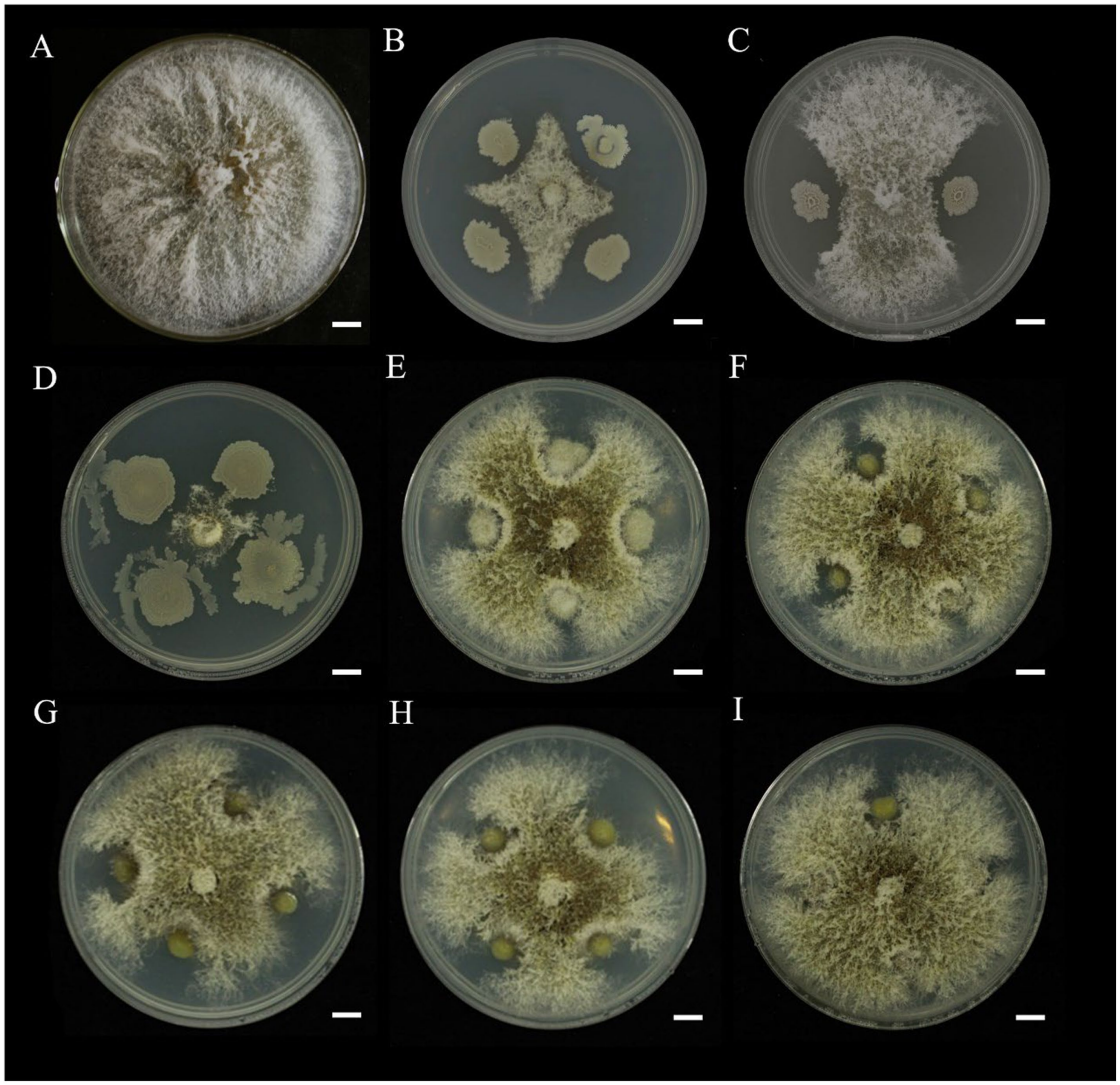
Screening of antagonistic bacteria against the pathogen. **(A)** Control (CK); **(B)** HN2-9 in a four-point confrontation assay (67.55%); **(C)** HN2-9 in a two-point confrontation assay; **(D)** HN1-20 (74.13%); **(E)** HN2-3 (34.78%); **(F)** HN2-6 (34.02%); **(G)** HN2-11 (42.19%); **(H)** HN2-16 (40.30%); and **(I)** HN3-9 (31.96%). Values in parentheses indicate the inhibition rate of each bacterial strain. Scale bar = 1 cm.

A total of 79 bacterial isolates were obtained from rhizosphere soil of healthy asparagus plants collected from stem blight–affected fields and were purified and preserved for further analysis. Using a dual-culture plate confrontation assay, seven isolates exhibited antagonistic activity against the stem blight pathogen ZF-C. When the mycelium of the control group completely covered the PSA plates, varying degrees of growth inhibition were observed among the tested strains (Figure 1).

Among these antagonistic isolates, strain HN1-20 exhibited the highest inhibition rate (74.13%, Figure 1D); however, its antagonistic performance was unstable in subsequent repeated assays. In contrast, strain HN2-9 consistently produced a clear and stable inhibition zone against the pathogen (Figures 1B,C), with an inhibition rate of 67.55%, indicating strong and reproducible antagonistic activity. Therefore, strain HN2-9 was selected for further detailed characterization and subsequent experiments.

**Figure 2:**
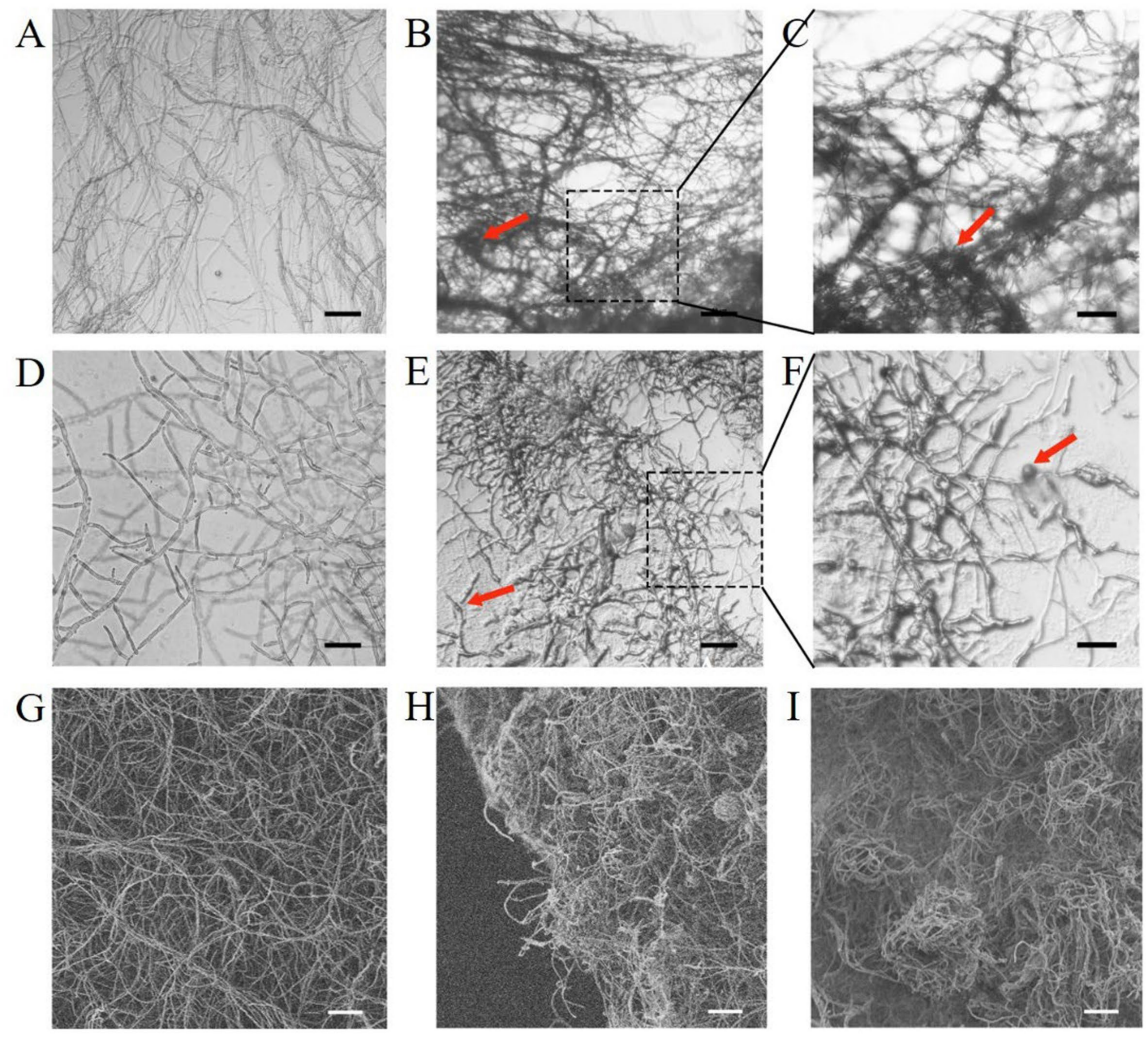
Morphological alterations of pathogen hyphae induced by strain HN2-9 observed by light microscopy and scanning electron microscopy. **(A–F)** Morphology of pathogen hyphae treated with strain HN2-9 as observed by light microscopy. **(A,D)** Hyphae of the control group (CK) showing normal morphology; **(B,E)** hyphae exhibiting shrinkage and twisting after treatment with strain HN2-9; **(C)** hyphae showing lysis and fragmentation; **(F)** vesiculation of hyphae induced by strain HN2-9. Scale bars = 50 μm for **A**, **B**, **D**, and **E**; 20 μm for **C** and **F**. **(G–I)** Scanning electron microscopy images of pathogen hyphae. **(G)** Hyphae of the control group (CK) with intact morphology; **(H,I)** hyphae treated with strain HN2-9 showing severe structural deformation. Scale bars = 20 μm.

Microscopic observation revealed clear morphological differences in pathogen hyphae after interaction with HN2-9 (Figure 2). In the control group treated with sterile water, hyphae of strain ZF-C were smooth, sparsely branched, and exhibited regular dendritic growth with clear spacing between hyphae (Figures 2A,D). In contrast, when co-cultured with HN2-9, pathogen hyphae showed severe growth abnormalities, including thinning, shrinkage, irregular branching, and entanglement (Figures 2B,C). Vesicle-like swelling was observed at the hyphal tips (Figure 2E), and in some cases, hyphae exhibited breakage or dissolution (Figure 2F).

Scanning electron microscopy further confirmed these observations. Compared with the control (Figure 2G), hyphae in the treatment group were highly curled, twisted, and aggregated into clumps, and no directional growth toward strain HN2-9 was observed (Figure 2H). Surface erosion and fragmentation of hyphae were evident (Figure 2I), suggesting that strain HN2-9 inhibited pathogen growth mainly through the secretion of antifungal metabolites and/or enzymatic substances that disrupted hyphal integrity. These results further demonstrated the strong antagonistic effect of HN2-9 against the stem blight pathogen.

Strain HN2-9 formed pale yellow, semi-transparent colonies on LB and PSA media. On LB medium, colonies were raised with wrinkled, rough, and moist surfaces and irregular edges (Supplementary Figure S2A), whereas colonies on PSA medium were smooth, moist, and circular with regular margins (Supplementary Figure S2B). Gram staining revealed that strain HN2-9 was a Gram-positive rod-shaped bacterium (Supplementary Figure S2C). In addition, when cultured on starch-containing LB medium followed by iodine staining, a distinct clear hydrolysis zone was observed surrounding the colonies (Supplementary Figure S2D), indicating the strong starch-degrading (amylolytic) activity of strain HN2-9.

For molecular identification, the 16S rDNA gene of strain HN2-9 was amplified and sequenced, yielding a fragment of 1,482 bp. BLAST analysis showed that the 16S rDNA sequence of HN2-9 shared the highest similarity (99.53%) with *Bacillus amyloliquefaciens* strain HK1 (CP018902.1), indicating that HN2-9 belongs to the species *B. amyloliquefaciens* (Supplementary Figure S2E). Furthermore, phylogenetic analysis based on the *gyrA* gene showed that strain HN2-9 clustered closely with reference strains of *B. amyloliquefaciens* (Supplementary Figure S2F), further supporting its taxonomic assignment to this species.

Combined with colony morphology and physiological and biochemical characteristics, strain HN2-9 was conclusively identified as *B. amyloliquefaciens*.

### Growth curve and condition optimization

**Figure 3:**
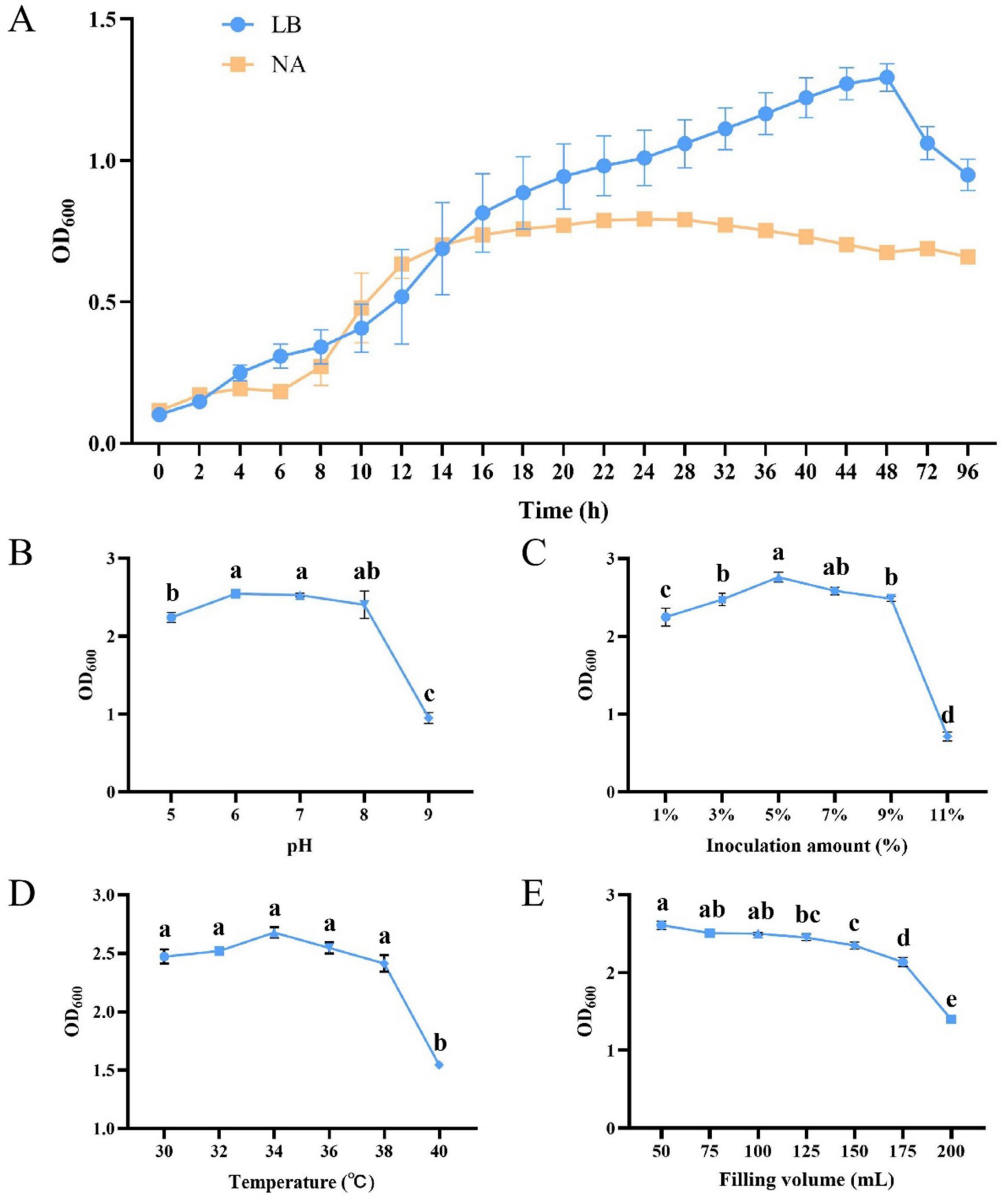
Growth characteristics of antagonistic bacterium strain HN2-9. **(A)** Growth curve of HN2-9. **(B–E)** Effects of different pH values, inoculum sizes, temperatures, and culture volumes on the growth activity of HN2-9, respectively. Data are presented as mean ± SD of three biological replicates. Different letters indicate significant differences among treatments according to Tukey’s multiple comparison test (*p* < 0.05).

The growth curves of HN2-9 in LB and NA media are shown in Figure 3A. Strain HN2-9 was able to grow in both media, while consistently exhibiting higher OD_600_ values in LB than in NA throughout the cultivation period. In LB medium, OD_600_ increased continuously during the first 24 h, followed by a gradual increase to a maximum at approximately 48 h, after which a decline was observed. In contrast, growth in NA medium increased more slowly and reached a relatively stable level after 24–32 h, with only slight fluctuations thereafter. Based on the overall growth trend in LB medium, cultures incubated for 20 h were selected as seed inoculum for subsequent experiments.

The effects of pH, inoculum size, temperature, and medium volume on HN2-9 growth are summarized in Figures 3B–E. The highest growth was observed at pH 6–7, which did not differ significantly from that at pH 8. Among the inoculum sizes tested, 5% produced the highest OD_600_ value, although no significant difference was detected between the 5 and 7% treatments. Growth remained relatively stable from 30 °C to 38 °C, with all treatments showing comparable OD_600_ values, whereas growth decreased significantly at 40 °C. Medium volume had a significant effect on growth, with the highest OD_600_ recorded at 50 mL and a gradual decline observed as the culture volume increased. Based on these results, HN2-9 was cultured at 34 °C and 200 r/min for 24 h in 50 mL LB medium at pH 7 with a 5% inoculum for subsequent experiments.

### Shake flask fermentation and medium component optimization

A 1% (v/v) inoculum of strain HN2-9 was introduced into 100 mL of LB medium and cultured at 30 °C with shaking at 200 r/min for 5 d to evaluate its baseline antifungal activity. The resulting sterile fermentation filtrate was used to prepare amended agar plates. Under these fixed conditions, the fermentation filtrate exhibited strong inhibitory activity against ZF-C, with an inhibition rate of 35.76% ± 2.04% (Supplementary Figure S3). At this time point, the mycelia of the pathogen in the control treatment had completely covered the plate.

**Figure 4:**
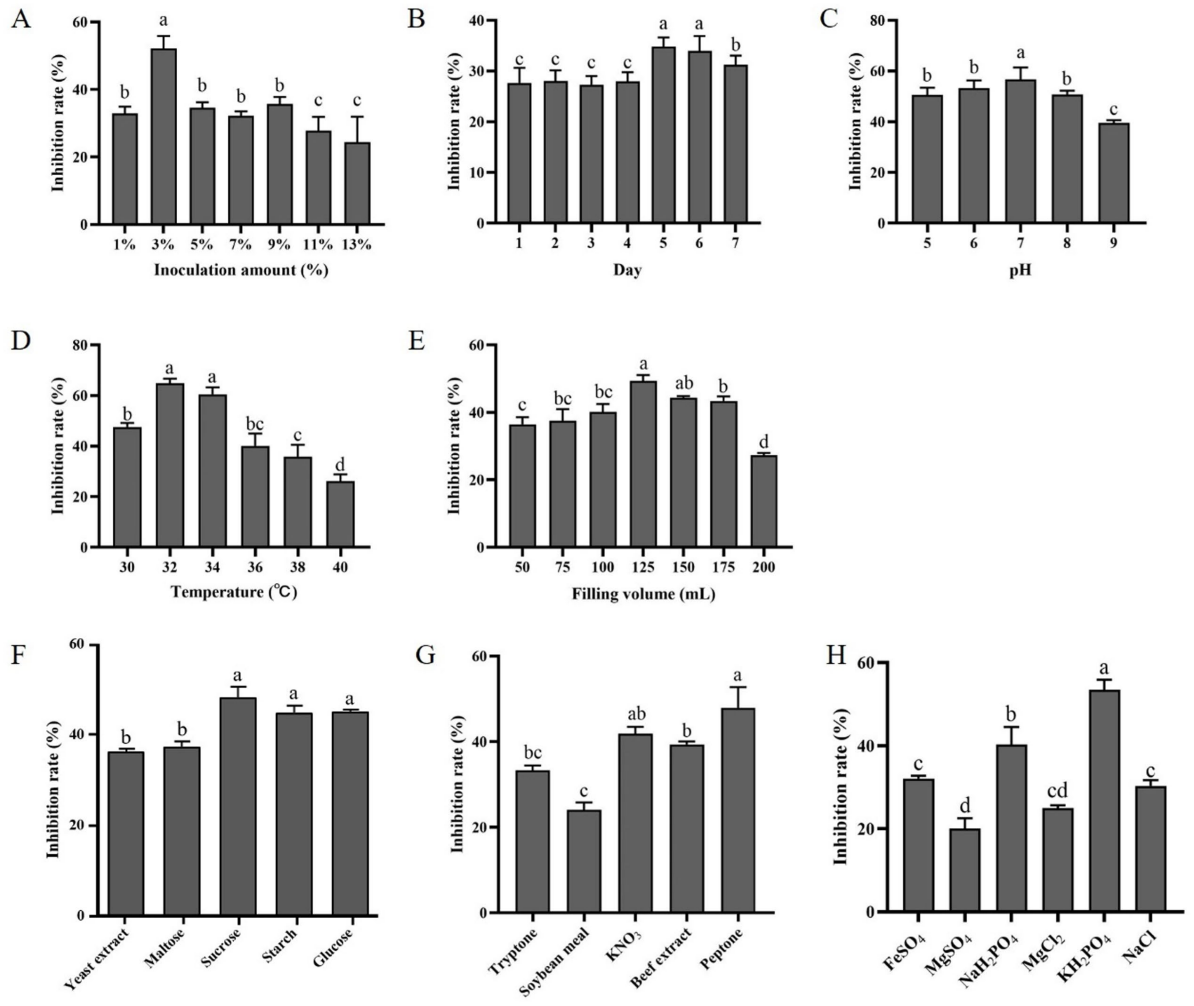
Effects of fermentation conditions and medium composition on the antibacterial activity of antagonistic strain HN2-9. **(A)** Effect of different inoculum sizes on inhibition rate; **(B)** Effect of different shaking culture durations on inhibition rate; **(C)** Effect of medium pH on inhibition rate; **(D)** Effect of incubation temperature on inhibition rate; **(E)** Effect of culture volume on inhibition rate; **(F–H)** Effects of different medium components on the antibacterial activity of fermentation filtrates, including different carbon sources **(F)**, nitrogen sources **(G)**, and inorganic salts **(H)**. Data are presented as mean ± SD of three biological replicates. Different letters indicate significant differences among treatments according to Tukey’s multiple comparison test (*p* < 0.05).

To further enhance the antifungal activity of the fermentation filtrate, key fermentation parameters were subsequently optimized using single-factor shake flask experiments. The antifungal activity varied with inoculum size, incubation time, initial pH, temperature, and medium volume. The inhibition rate increased with inoculum size and reached a maximum of 52.14% at a 3% inoculum. A five-day incubation period yielded the highest inhibitory activity, whereas longer incubation times resulted in a decline in inhibition. The optimal initial pH and temperature were 7 (56.69%) and 32 °C (64.85%), respectively, and the highest inhibition rate (49.39%) was obtained with a medium volume of 125 mL (Figures 4A–E).

Based on these results, the optimal shake flask fermentation conditions were determined to be a 3% inoculum in 125 mL LB medium at pH 7, incubated at 32 °C and 200 r/min for 5 days.

**Figure 5:**
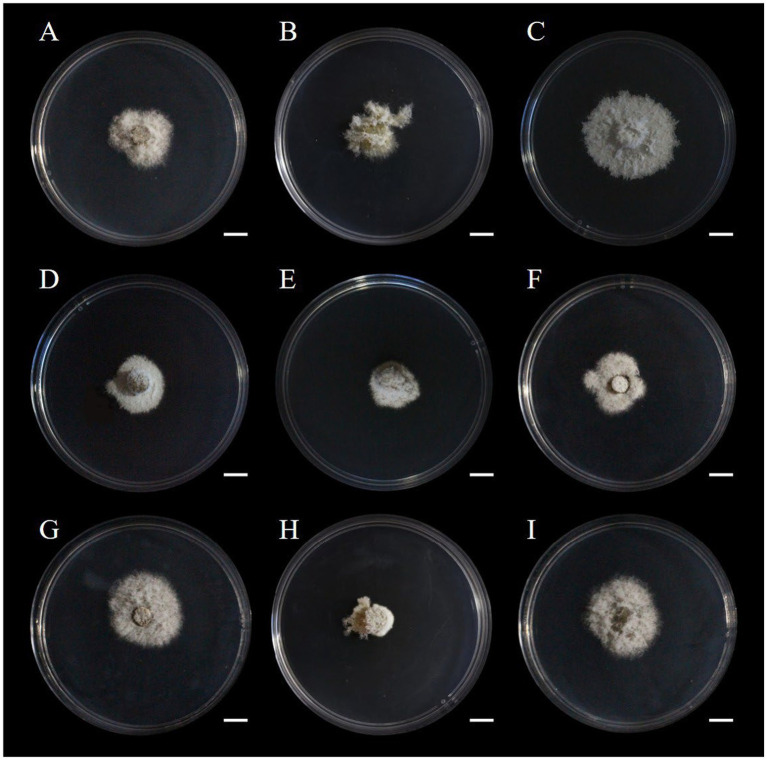
Plate inhibition assay results of fermentation filtrates from different treatments in the orthogonal experimental design. **(A–I)** Antibacterial activities of fermentation filtrates corresponding to treatments 1–9 in Supplementary Table S1.

Single-factor optimization of medium components indicated that sucrose (48.09%) was the best carbon source, peptone (47.89%) the best nitrogen source, and KH_2_PO_4_ (53.51%) the optimal inorganic salt (Figures 4F–H). Using these results, an L9 orthogonal design was applied to optimize carbon, nitrogen, and inorganic salt levels. The optimal combination was 0.7% sucrose, 1% KH_2_PO_4_, and 0.5% peptone, which increased the inhibition rate against ZF-C to 92.07% ± 2.67% (Figure 5, Supplementary Tables S1, S2). Range analysis indicated the influence order of the three factors as KH_2_PO_4_ > peptone > sucrose.

### Effect of HN2-9 fermentation filtrate on spore germination of ZF-C

HN2-9 fermentation filtrate significantly inhibited ZF-C spore germination (Supplementary Table S3). At 18 h, the control spore germination rate was 55.72%, whereas treated spores were 28.48%. Between 36 and 72 h, treated spores reached only 37.51%, with hyphal growth markedly reduced, demonstrating effective suppression of both spore germination and hyphal extension.

### Effects of HN2-9 treatments on asparagus seed germination

To evaluate whether different HN2-9 preparations affect asparagus seed germination, seeds were treated with HN2-9 fermentation broth, fermentation filtrate, 10^−1^ and 10^−2^ dilutions, or sterile water. Germination rates were recorded from day 5 to day 10. No significant differences were observed among treatments, indicating that HN2-9 fermentation products neither inhibited nor promoted seed germination (Supplementary Figure S4). On day 10, germination rates were 90 ± 4%, 89.33 ± 2.31%, 88.00 ± 2%, 90.67 ± 5.03%, and 90.00 ± 2% for the fermentation broth, filtrate, 10^−1^, 10^−2^, and water control, respectively (Supplementary Figure S4).

### Biocontrol efficacy of HN2-9 on asparagus stems

**Figure 6:**
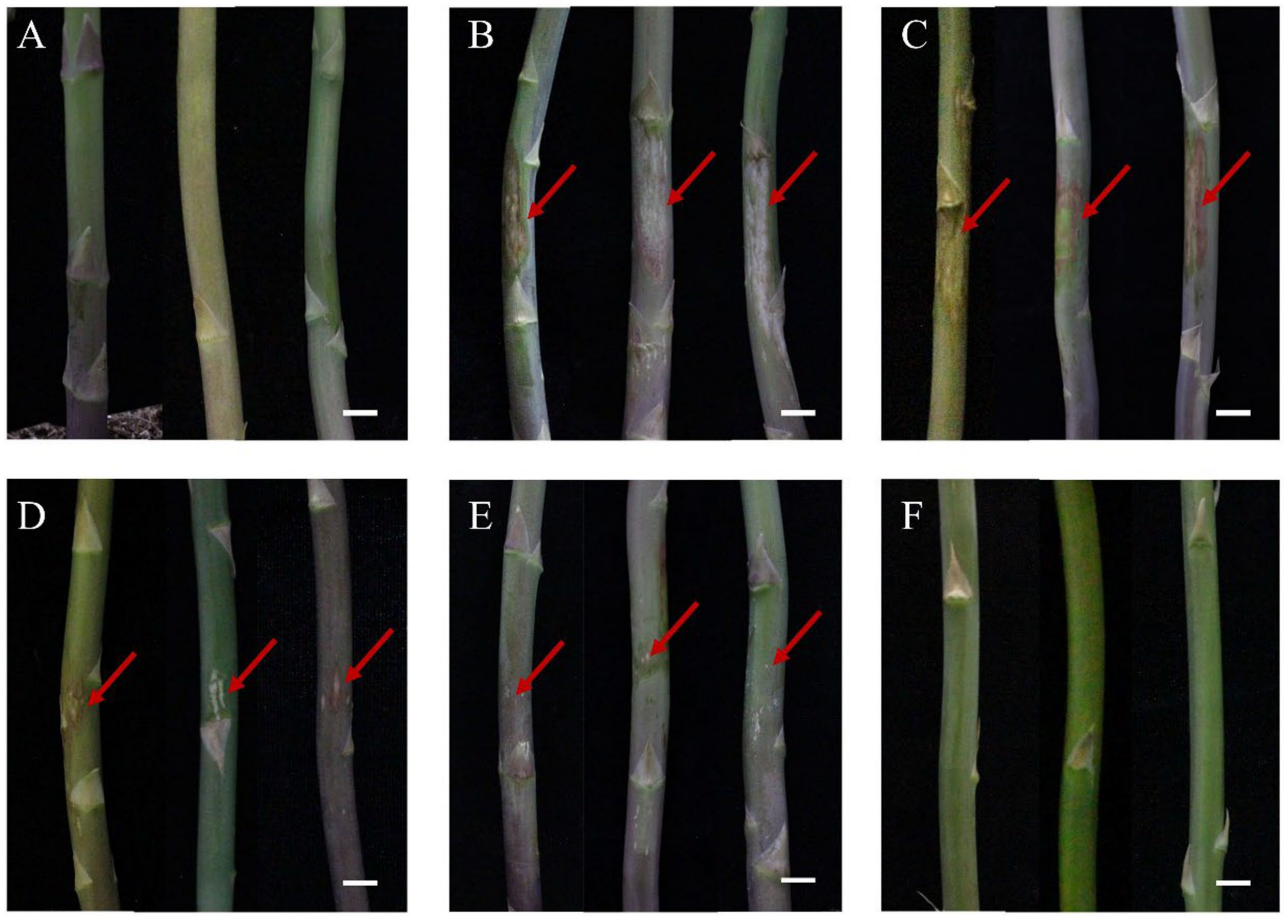
Evaluation of disease resistance on asparagus stems. **(A)** Uninoculated liquid medium treatment; **(B)** inoculation with pathogen spore suspension alone; **(C)** spore suspension supplemented with HN2-9 at OD_600_ = 0.8; **(D)** spore suspension supplemented with HN2-9 at OD_600_ = 1.6; **(E)** spore suspension supplemented with HN2-9 at OD_600_ = 2.0; **(F)** HN2-9 treatment alone. Scale bar = 1 cm. Arrows indicate the symptomatic lesions at the inoculation sites.

The protective effect of HN2-9 on asparagus stems was further evaluated. Seven days after inoculation with ZF-C spore suspension, stems treated with the pathogen alone exhibited severe symptoms, with a disease index of 72.5 ± 3.56, whereas water-treated control stems showed no lesions (Figure 6; Supplementary Table S4). Co-application of HN2-9 at different concentrations with ZF-C significantly reduced stem disease severity, with smaller lesion size and length compared to the pathogen-only treatment. Disease suppression increased with HN2-9 concentration: at OD_600_ = 2.0, the disease index decreased to 20 ± 5.41, corresponding to a control efficacy of 72.41% (Supplementary Table S4).

Application of HN2-9 alone had no adverse effects on garden asparagus stems; plants grew normally without lesions or wilting (Figure 6). These results demonstrate that HN2-9 effectively suppresses garden asparagus stem blight without harming the host, supporting its potential use as a safe and effective biocontrol agent for further integrated disease management studies.

## Discussion

Asparagus stem blight poses a significant threat to asparagus production, causing substantial yield losses and economic impact for growers. This study provides a systematic evaluation of a *B. amyloliquefaciens* strain (HN2-9) isolated from asparagus rhizosphere soil for the control of asparagus stem blight, a disease for which biological control studies remain limited, and highlights its strong potential as a locally adapted biocontrol agent. The pathogen ZF-C, isolated from infected asparagus plants, was identified as *P. asparagi* based on morphological characteristics and ITS sequence analysis. This disease is widespread across China, and previous studies have reported that isolates from different regions vary in biological traits and genetic diversity, with FJ2 exhibiting the highest virulence among six provincial isolates (Yang et al., 2020). Our work focused solely on the ZF-C isolate, and further investigation is required to assess whether biocontrol strategies are equally effective against isolates from other regions. Understanding regional variability is crucial for developing broadly applicable biocontrol approaches, as pathogen adaptation may influence both infection dynamics and the efficacy of antagonistic agents.

The antagonistic bacterium HN2-9 was identified as *B. amyloliquefaciens* through 16S rDNA and the conserved *gyrA* gene, combined with morphological observations and physiological-biochemical characterization. Members of this species are known to promote plant growth and suppress pathogens through secondary metabolites, including lipopeptides, hydrolytic enzymes, and potentially volatile organic compounds (VOCs) (Grahovac et al., 2023; Kai et al., 2009). Although the exact metabolites secreted by HN2-9 were not characterized in this study, the fermentation filtrate demonstrated clear inhibitory effects against ZF-C hyphal growth, indicating that extracellular factors are involved in the antagonistic activity of HN2-9.

Optimization of fermentation conditions markedly enhanced the inhibitory effect of HN2-9. Initial fermentation filtrates inhibited ZF-C by only 35.76%, whereas after systematic optimization—including adjustment of pH, temperature, inoculum size, culture volume, and replacement of medium components with sucrose, peptone, and KH₂PO₄—followed by orthogonal design analysis, inhibition increased to 92.07%. Although both KH_2_PO_4_ and NaH_2_PO_4_ provide phosphate and may exert similar buffering effects, KH_2_PO_4_ resulted in stronger antagonistic activity. This difference may be associated with the distinct physiological roles of K^+^ and Na^+^, which can influence bacterial growth, metabolism, and the production of antagonistic compounds. It should be noted, however, that the optimal conditions for antifungal activity did not always coincide with those that favored bacterial growth. For example, growth was numerically highest at a 5% inoculum size and 34 °C, whereas maximal antifungal activity was obtained at a 3% inoculum size and 32 °C. Similarly, lower culture volumes promoted bacterial growth, while a culture volume of 125 mL yielded stronger antagonistic activity. These observations suggest that enhanced antagonistic activity is not solely attributable to increased bacterial biomass. Other physiological responses associated with culture conditions may also contribute to antagonistic performance, although the underlying mechanisms were not investigated in the present study. Consistent with this interpretation, optimization of medium composition substantially affected the inhibitory activity of strain HN2-9. Range analysis revealed that KH₂PO₄ exerted the greatest influence on inhibitory activity, followed by peptone and sucrose. These findings indicate that the antagonistic activity of strain HN2-9 is highly sensitive to culture conditions. Similar effects have been reported in *Bacillus* species, where changes in medium composition and fermentation parameters influence the production of antifungal metabolites (Zhao et al., 2014; Qiu et al., 2014).

*In vitro* assays demonstrated that culture filtrates of HN2-9 strongly inhibited fungal growth, and greenhouse experiments further confirmed significant reductions in disease severity. These findings are consistent with previous reports that many biocontrol bacteria produce secrete antimicrobial compounds that inhibit pathogen growth and virulence *in vitro* and in planta (Bonaterra et al., 2022). The strong antifungal activity observed in plate assays indicates that diffusible extracellular factors may play an important role in the antagonistic activity. In addition, greenhouse experiments were conducted using live bacterial inocula rather than culture filtrates, as effective biocontrol generally relies on multiple factors beyond antimicrobial metabolites alone. Live bacterial formulations are commonly used in biocontrol studies because they can maintain continuous production of bioactive compounds under environmental conditions and may provide more stable disease suppression compared with cell-free filtrates. Such advantages have been reported in previous studies where live inocula demonstrated stronger and more sustained biocontrol efficacy than filtrates in planta (Riseh et al., 2025). For instance, bacterial isolates applied as live inocula have been shown to confer effective disease suppression in greenhouse systems (Zenelt and Krawczyk, 2025).

Several limitations should be acknowledged. This study was conducted under greenhouse conditions, which may not fully reflect field complexity, including environmental fluctuations, native microbial communities, and pathogen pressure. In addition, no comparative evaluation with commercial biocontrol agents or fungicides was included, limiting direct benchmarking of efficacy. Furthermore, the antagonistic mechanisms of HN2-9 were also not experimentally validated in this study. Although the culture filtrate exhibited strong antifungal activity against *P. asparagi*, the active substances responsible for pathogen inhibition remain unknown. Therefore, the proposed involvement of lipopeptides, hydrolytic enzymes, VOCs, or other antimicrobial metabolites remains speculative. Future studies should focus on mechanistic characterization of the antagonistic activity, including antifungal metabolite profiling, lipopeptide-related gene detection, enzyme activity assays, or VOC analysis, benchmark comparisons with commercial biocontrol products and/or fungicides, and field validation of HN2-9 to better contextualize its performance under practical disease management conditions.

In conclusion, *B. amyloliquefaciens* HN2-9 exhibited strong inhibitory activity against *P. asparagi* ZF-C, and optimization of fermentation conditions significantly enhanced its biocontrol efficacy. The strain effectively reduced disease severity in greenhouse trials, demonstrating potential as a safe and environmentally sustainable tool for managing asparagus stem blight. Future work should focus on elucidating the active metabolites and assessing field-level efficacy to support practical, large-scale application.

## Data Availability

The original contributions presented in the study are included in the article/Supplementary material, further inquiries can be directed to the corresponding authors.
