# Supplementary material for: Isolation and biocontrol potential of Bacillus amyloliquefaciens against asparagus stem blight
Source: Front Microbiol. 2026 Jul 9;17:1872041. doi: 10.3389/fmicb.2026.1872041 (PMC13391875; doi:10.3389/fmicb.2026.1872041)
Supplement: Supplementary file 1 [file Data_Sheet_1.docx]

**Table S1** Factor levels of medium components

| Level | Concentrations of different factors | | |
| --- | --- | --- | --- |
|  | Sucrose (%) | KH_2_PO_4_ (%) | Peptone (%) |
|  | A | B | C |
| 1 | 0.3 | 0.5 | 0.5 |
| 2 | 0.5 | 1 | 1 |
| 3 | 0.7 | 1.5 | 1.5 |

**Table S2** Analysis table of orthogonal optimization test for each component

| Treatment | Factor A | Factor B | Factor C | Inhibition rate (%) |
| --- | --- | --- | --- | --- |
| 1 | 1 | 1 | 1 | 81.87±1.37 |
| 2 | 1 | 2 | 2 | 83.31±1.89 |
| 3 | 1 | 3 | 3 | 68.74±2.38 |
| 4 | 2 | 1 | 2 | 85.59±1.03 |
| 5 | 2 | 2 | 3 | 88.68±1.61 |
| 6 | 2 | 3 | 1 | 79.16±1.92 |
| 7 | 3 | 1 | 3 | 70.46±3.06 |
| 8 | 3 | 2 | 1 | 92.07±2.67 |
| 9 | 3 | 3 | 2 | 73.05±2.75 |
| K1 | 233.92 | 237.92 | 253.10 |  |
| K2 | 253.43 | 264.06 | 241.95 |  |
| K3 | 235.58 | 220.95 | 227.88 |  |
| k1 | 77.97 | 79.31 | 84.37 |  |
| k2 | 84.48 | 88.02 | 80.65 |  |
| k3 | 78.53 | 73.65 | 75.96 |  |
| R | 6.51 | 14.37 | 8.41 |  |
| Order of influence | B＞C＞A | | |  |
| Optimal level | A_3_ | B_2_ | C_1_ |  |
| Optimal combination | A_3_B_2_C_1_ | | |  |

**Table S3** Effect of HN2-9 fermentation filtrate on spore germination of ZF-C

| Treatment time (h) | Spore germination rate (%) | |
| --- | --- | --- |
|  | CK | HN2-9 fermentation filtrate |
| 18 | 55.72±4.18^a^ | 28.48±5.36^b^ |
| 21 | 63.29±2.57^a^ | 32.74±3.05^b^ |
| 24 | 74.13±1.87^a^ | 35.60±2.42^b^ |
| 36 | 81.47±3.64^a^ | 37.51±2.93^b^ |
| 48 | 83.06±3.71^a^ | 37.51±2.93^b^ |
| 60 | 83.06±3.71^a^ | 37.51±2.93^b^ |
| 72 | 83.06±3.71^a^ | 37.51±2.93^b^ |

Note: Values are presented as mean ± standard deviation (SD) (n = 3). Statistical differences between the CK and HN2-9 fermentation filtrate treatments at each time point were analyzed using Student’s t-test. Different letters (a, b) within the same time point indicate significant differences between treatments at P < 0.05.

**Table S4** Effects of different concentrations of HN2-9 on the control of asparagus stem blight.

| Concentration of HN2-9(OD_600_) | Disease index | Relative efficacy |
| --- | --- | --- |
| CK | 72.5±3.56^a^ |  |
| 0.8 | 57.5±4.13^b^ | 20.69% |
| 1.6 | 42.5±2.65^c^ | 44.83% |
| 2.0 | 20±5.41^d^ | 72.41% |

Note: Data are presented as mean ± standard deviation. The experiment was independently repeated three times, with at least 5 plants per treatment in each replicate. Statistical analysis was performed using one-way analysis of variance (ANOVA) followed by Tukey’s multiple comparison test. Different letters indicate significant differences among treatments at P < 0.05.


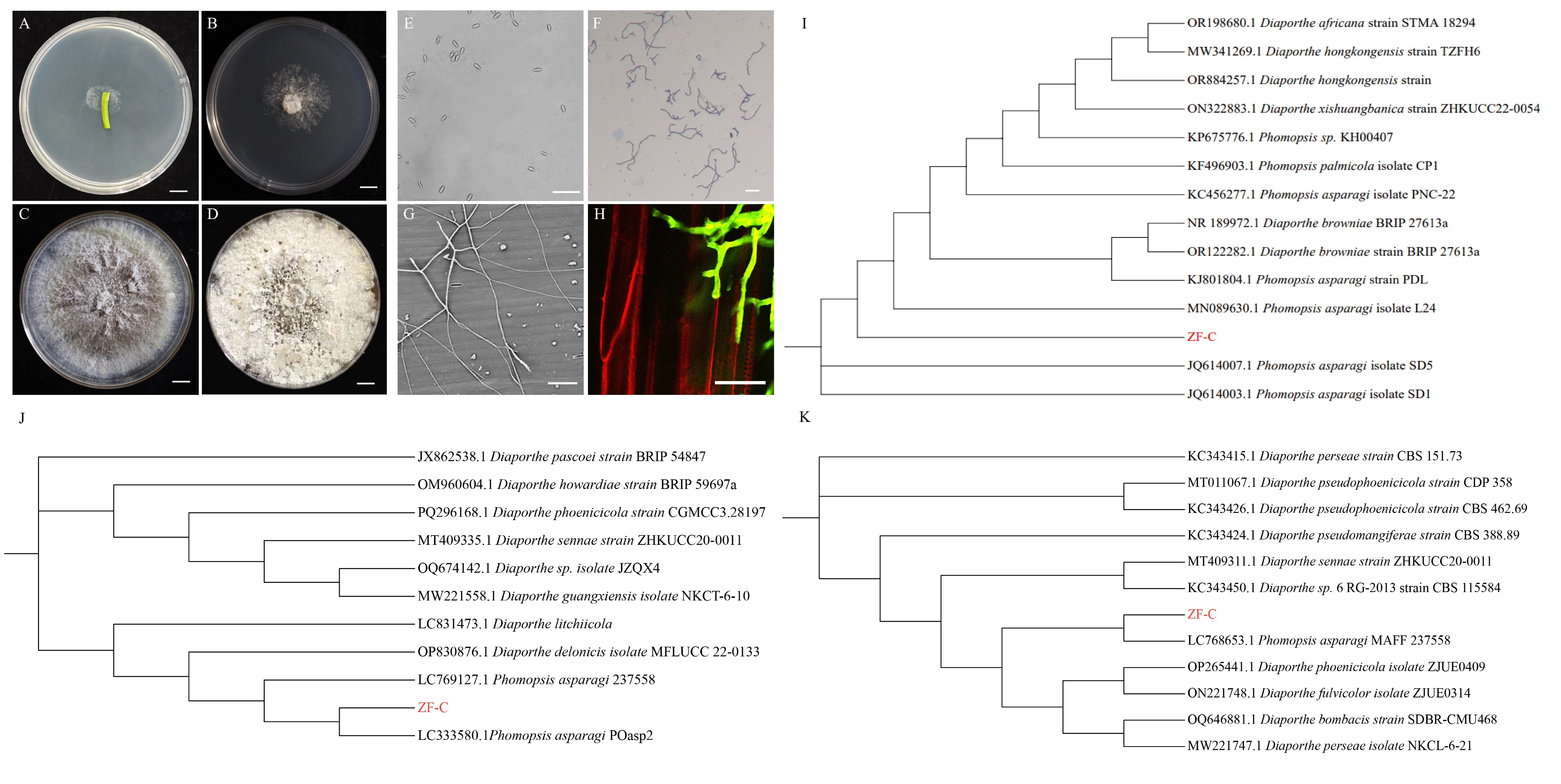


**Fig. S1** Isolation and identification of the pathogen causing asparagus stem blight.

(A–D) Isolation and cultural characteristics of the pathogen. (A) Isolation of the pathogen from symptomatic asparagus stems. (B) Early mycelial growth. (C) Late hyphal growth. (D) Conidia production. Bars = 1 cm. (E–H) Microscopic observations of the pathogen. (E) Conidia morphology. (F) Mycelial morphology under light microscopy. (G) Mycelial growth on the surface of asparagus stems. (H) Confocal microscopy of mycelial infection. Asparagus cell walls were stained red with propidium iodide (PI), and pathogen hyphae were stained green with WGA-AF488. Bars = 20 μm. (I–K) Phylogenetic tree of the pathogen based on internal transcribed spacer (ITS), TEF1-α and CAL sequences.


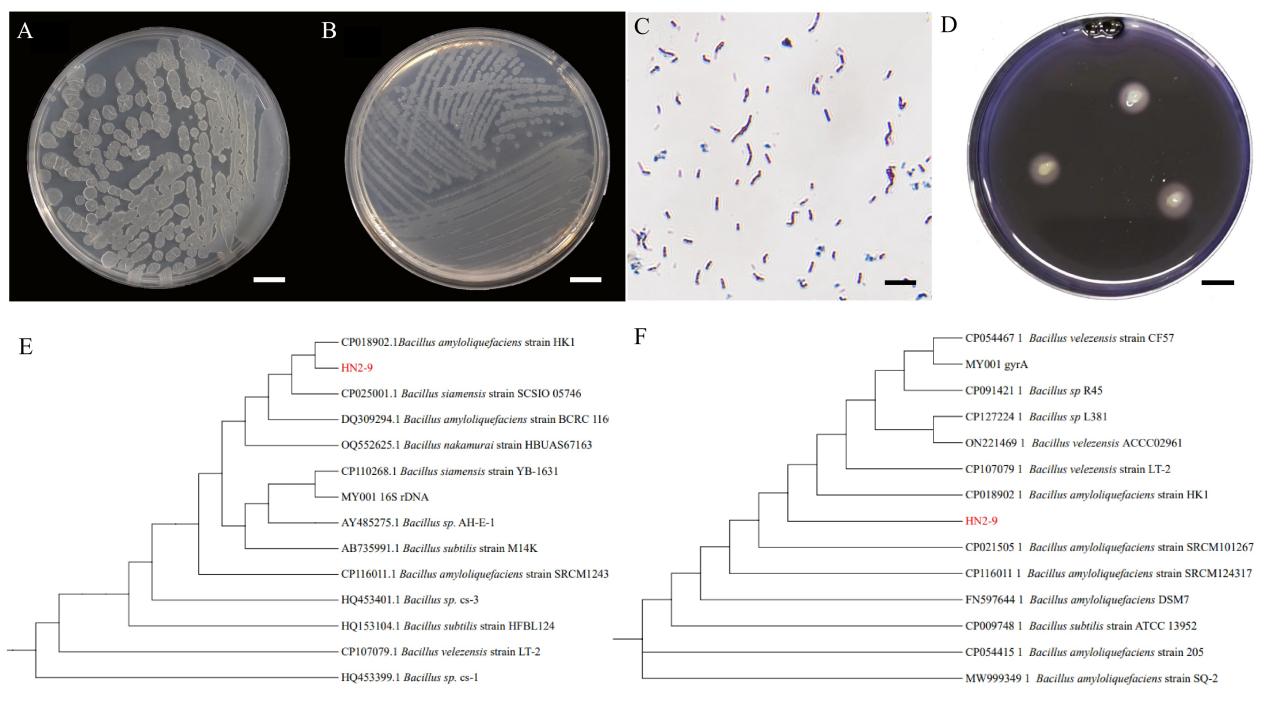


**Fig. S2** Identification and characterization of antagonistic bacterium strain HN2-9.

(A, B) Colony morphology of strain HN2-9 grown on LB and PSA media, respectively. Scale bars = 1 cm. (C) Gram staining of strain HN2-9 observed under a light microscope. Scale bar = 10 μm. (D) Starch hydrolysis assay of strain HN2-9 showing a clear halo around the colony after iodine staining, indicating amylase activity. Scale bar = 1 cm. (E) Phylogenetic tree of strain HN2-9 constructed based on 16S rDNA sequences. (F) Phylogenetic tree of strain HN2-9 constructed based on gryA sequences.


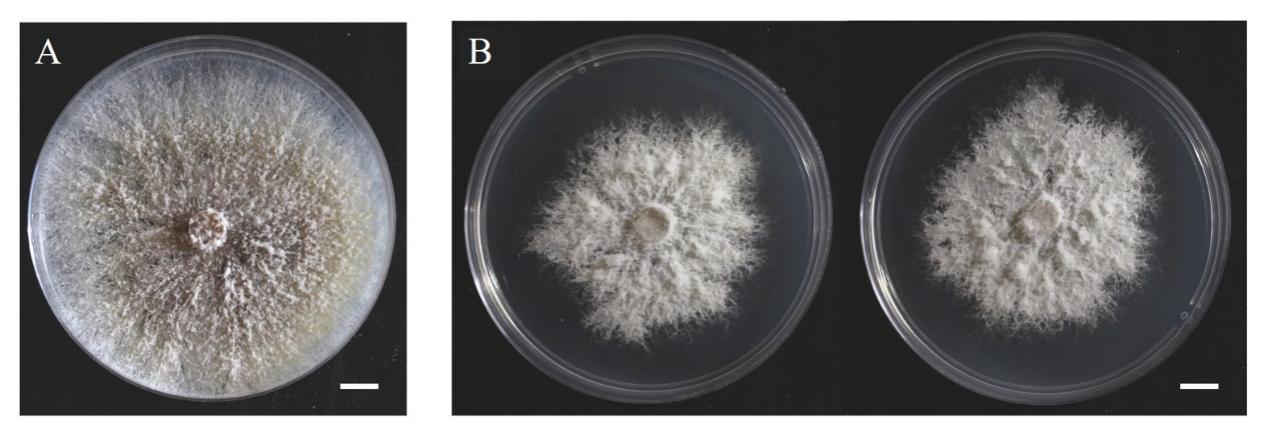


**Fig. S3** Inhibitory effect of the HN2-9 fermentation filtrate against the pathogen.

(A) Mycelial growth of the pathogen on the control plate; (B) Mycelial growth on plates amended with the fermentation filtrate. Bars = 1 cm.


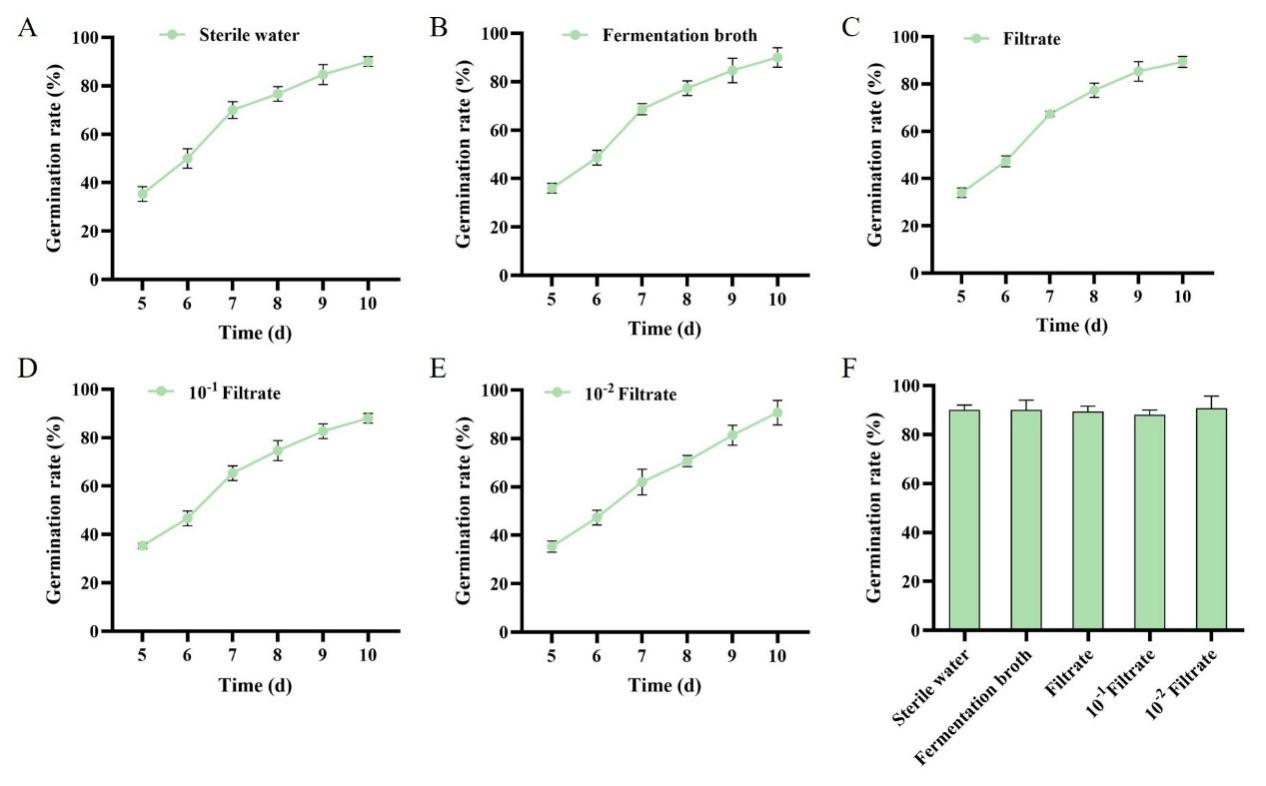


**Fig. S4** Effects of different HN2-9 treatment solutions on garden asparagus seed germination. (A) Sterile water (control); (B) fermentation broth; (C) fermentation filtrate; (D) 10⁻¹ diluted fermentation filtrate; (E) 10⁻² diluted fermentation filtrate; (F) seed germination rate of each treatment on day 10.
